# Supplementary material for: Development and Validation of the Chinese Frailty Screening Scale: A Study among Community-Dwelling Older Adults in Shanghai
Source: Int J Environ Res Public Health. 2022 Sep 19;19(18):11811. doi: 10.3390/ijerph191811811 (PMC9517433; doi:10.3390/ijerph191811811)
Supplement: Supplementary file 1 [file ijerph-19-11811-s001.zip › ijerph-1857008-supplementary.pdf]

Supplementary Materials

Table S1. The initial 33 items of the Chinese frailty screening scale (CFSS)

	Items
**Illnesses**	
a1	您是否患有5种及以上的慢性疾病？（如：高血压；血脂异常；糖尿病或血糖升高；癌症等恶性肿瘤(不包括轻度皮肤癌)；慢性肺部疾病；肝脏疾病；心肌梗塞、冠心病、心绞痛、充血性心力衰竭或其他心脏病；中风；肾脏疾病；胃部或其它消化系统疾病；阿尔兹海默病或帕金森病；关节炎或风湿病；哮喘）<br>Have you been diagnosed with at least 5 illnesses by doctors? (i.e., Hypertension; Dyslipidemia; Diabetes or high blood sugar; Cancer or malignant tumor (excluding minor skin cancers); Chronic lung diseases; Liver disease; Heart attack, coronary heart disease, angina, congestive heart failure, or other heart problems; Stroke; Kidney disease; Stomach or other digestive disease; Alzheimer's or Parkinson's disease; Arthritis or rheumatism; Asthma)
a2	您是否服用 3 种及以上的处方药？<br>Do you take ≥3 prescription drugs?
a3	最近一周，您有没有出现尿频、尿急、尿失禁的情况？<br>In the past week, have you experienced frequent urination, urgency or incontinence?
a4	您有没有经常受到身体疼痛的困扰？<br>Are you often troubled with any body pains?
a5	最近一个月，您在睡眠方面有没有困难？比如难以入睡，夜里经常醒来，或早上过早地醒来。<br>In the last month, do you have difficulty with sleeping, such as having trouble falling asleep, waking up frequently during the night, or waking up too early in the morning?
**Energy**	
a6	您经常出现嗜睡、头昏眼花的情况吗？<br>Do you have problem with drowsiness or dizziness?
a7	您经常出现胸闷、呼吸短促的情况吗？<br>Do you have problem with chest distress or shortness of breath?
a8	最近一个月，您有没有经常感觉到疲倦？<br>Did you often feel tired or fatigue in the last month?
a9	您经常锻炼身体吗？<br>Do you often do physical exercise?
**Nutrition**	
a10	最近三个月，您的进食量有没有减少（由于食欲减退、消化不良、牙口不好或吞咽困难）？<br>In the last three months, did you eat less due to loss of appetite, indigestion, teeth problem or dysphagia?
a11	最近一年，在非有意减肥的情况下，您的体重有没有减轻超过 9 斤（4.5kg）或降低超过 5%？

	In the last year, have you lost more than 9 jin (4.5kg) or at least 5% of previous year's body weight unintentionally (i.e., not due to dieting or exercise)
**Sensory function**	
a12	您的生活有没有因为视力问题而受到影响？<br>Do you experience problems in your daily life due to poor vision?
a13	您的生活有没有因为听力问题而受到影响？<br>Do you experience problems in your daily life due to poor hearing?
**Strength**	
a14	您提 10 斤（5kg）重的东西，有困难吗？（如一袋大米）<br>Do you have difficulty with lifting or carrying weights over 10 jin (5kg), like a heavy bag of groceries?
a15	您将手臂抬高到与肩平齐有没有困难？<br>Do you have difficulty with reaching or extending your arms to shoulder level?
a16	您从桌上拿起一小枚硬币，有困难吗？<br>Do you have difficulty with picking up a small coin from a table?
a17	如果中途不休息，爬上十级台阶或一层楼，您觉得有没有困难？<br>Do you have difficulty with climbing 10 stairs or a flight without resting?
a18	您捡起地上的小物品有没有困难？<br>Do you have difficulty with picking up a small object from floor?
**Mobility**	
a19	您能独自出门吗？<br>Can you go out alone?
a20	您能不能从床上或者椅子上站起来？<br>Do you have difficulty with getting out of bed or chair?
a21	您可以不借助扶手或者墙壁自己上楼梯吗？<br>Can you go upstairs without the help of handles or walls?
a22	您会因为害怕摔倒而放慢了脚步或做出其他调整吗？<br>Do you slow down by afraid of fall?
**Cognition**	
a23	您感觉您的记忆力有下降吗？<br>Do you think your memory has declined?
a24	您有没有经常忘记东西放在哪里或者忘记前一天发生的事情？<br>Do you often forget where things are or what happened the day before?
a25	您有没有经常走神或者难以集中注意力？<br>Do you often wander or have difficulty with concentrating?
a26	您是否注意到您的思考和推理能力发生了变化？<br>Do you feel that your skills in thinking and reasoning have been declining?
a27	您有无法判断日期/时间、方向/地点的情况吗？<br>Do you experience problems in identifying date/time or direction/place?
**Psychology**	
a28	您有对做什么事情都没兴趣而感到烦恼吗？<br>Are you bothered by lack of interests in doing anything?
a29	您对目前的生活满意吗？

	Are you satisfied with your current life?
a30	您觉得自己很没用吗？<br>Do you feel useless?
a31	您经常感到无助吗？<br>Do you often feel helpless?
a32	您喜欢呆在家里而不是出去做其他的事情吗？<br>Do you prefer to stay at home, rather than going out and doing new things?
a33	您经常感到紧张或焦虑吗？<br>Do you often feel nervous or anxious?

Table S2. The revised 20-item CFSS (CFSS-20) after expert consults

	**Items**
b1	您是否患有 5 种及以上的慢性疾病？（如：高血压；血脂异常；糖尿病或血糖升高；癌症等恶性肿瘤(不包括轻度皮肤癌)；慢性肺部疾病；肝脏疾病；心肌梗塞、冠心病、心绞痛、充血性心力衰竭或其他心脏病；中风；肾脏疾病；胃部或其它消化系统疾病；阿尔兹海默病或帕金森病；关节炎或风湿病；哮喘）<br>Have you been diagnosed with at least 5 illnesses by doctors? (i.e., Hypertension; Dyslipidemia; Diabetes or high blood sugar; Cancer or malignant tumor (excluding minor skin cancers); Chronic lung diseases; Liver disease; Heart attack, coronary heart disease, angina, congestive heart failure, or other heart problems; Stroke; Kidney disease; Stomach or other digestive disease; Alzheimer's or Parkinson's disease; Arthritis or rheumatism; Asthma)
b2	您有没有经常受到身体疼痛的困扰？<br>Are you often troubled with any body pains?
b3	最近一个月，您有没有经常感觉到疲倦？<br>Did you often feel tired or fatigue in the last month?
b4	最近三个月，您的进食量有没有减少（由于食欲减退、消化不良、牙口不好或吞咽困难）？<br>In the last three months, did you eat less due to loss of appetite, indigestion, teeth problem or dysphagia?
b5	最近一年，在非有意减肥的情况下，您的体重有没有减轻超过 9 斤（4.5kg）或降低超过 5%？<br>In the last year, have you lost more than 9 jin (4.5kg) or at least 5% of previous year’s body weight unintentionally (i.e., not due to dieting or exercise)
b6	您的生活有没有因为视力问题而受到影响？<br>Do you experience problems in your daily life due to poor vision?
b7	您的生活有没有因为听力问题而受到影响？<br>Do you experience problems in your daily life due to poor hearing?
b8	您提 10 斤（5kg）重的东西，有困难吗？（如一袋大米）<br>Do you have difficulty with lifting or carrying weights over 10 jin (5kg), like a heavy bag of groceries?
b9	如果中途不休息，爬上十级台阶或一层楼，您觉得有没有困难？<br>Do you have difficulty with climbing 10 stairs or a flight without resting?
b10	您能不能从床上或者椅子上站起来？<br>Do you have difficulty with getting out of bed or chair?
b11	您能不能连续行走 10 分钟或 400 米？<br>Do you have difficulty with walking 10 minutes or 400 meters continuously?
b12	最近一周，您有没有过连续行走 10 分钟或 400 米？<br>Have you done any walks for at least 10 minutes or 400 meters continuously in the last week?
b13	最近一周，您有没有出现尿频、尿急、尿失禁的情况？<br>In the past week, have you experienced frequent urination, urgency or incontinence?
b14	最近一个月，您在睡眠方面有没有困难？比如难以入睡，夜里经常醒来，或早上

	过早地醒来。<br>In the last month, do you have difficulty with sleeping, such as having trouble falling asleep, waking up frequently during the night, or waking up too early in the morning?
b15	最近一个月，您有没有经常忘记东西放在哪里或者忘记前一天发生的事情？<br>In the last month, did you often forget where things are or what happened the day before?
b16	最近一个月，您有没有经常走神或者难以集中注意力？<br>Did you often wander or have difficulty with concentrating in the last month?
b17	最近一个月，您有没有经常搞错日期、或者迷路？<br>Did you frequently get the date wrong or get lost in the last month?
b18	最近一个月，您有没有感觉做什么事情都不感兴趣？<br>Did you feel you were not interested in doing anything in the last month?
b19	最近一个月，您有没有总是情绪低落、高兴不起来？<br>Did you often feel depressed in the last month?
b20	最近一个月，您有没有担心一些不好的事情会发生在自己身上？<br>Were you afraid that something bad is going to happen to you in the last month?

Table S3. The results of logistic regression for the CFSS by different method (***sample 1***)

Item	Model 1		Model 2		Model 3	
	OR (95%CI)	P value	OR (95%CI)	P value	OR (95%CI)	P value
**b1**	**1.90 (1.32, 2.75)**	**0.001**	**1.96 (1.37, 2.82)**	**0.000**	**1.96 (1.37, 2.81)**	**0.000**
b2	1.02 (0.75, 1.38)	0.904				
**b3**	**2.75 (2.00, 3.76)**	**0.000**	**2.77 (2.07, 3.72)**	**0.000**	**2.75 (2.05, 3.69)**	**0.000**
**b4**	**1.67 (1.12, 2.49)**	**0.012**	**1.73 (1.18, 2.55)**	**0.005**	**1.69 (1.14, 2.49)**	**0.008**
b5	1.14 (0.67, 1.94)	0.623				
b6	1.14 (0.82, 1.59)	0.443			1.17 (0.84, 1.62)	0.362
b7	1.05 (0.74, 1.50)	0.780			1.07 (0.75, 1.52)	0.712
b8	1.13 (0.77, 1.66)	0.527				
**b9**	**1.93 (1.44, 2.60)**	**0.000**	**1.97 (1.48, 2.63)**	**0.000**	**1.93 (1.44, 2.59)**	**0.000**
b10	1.37 (0.91, 2.07)	0.132				
b11	0.79 (0.42, 1.51)	0.480				
**b12**	**2.42 (1.35, 4.35)**	**0.003**	**2.52 (1.87, 3.39)**	**0.000**	**2.54 (1.89, 3.42)**	**0.000**
b13	0.80 (0.55, 1.17)	0.243				
b14	1.15 (0.84, 1.56)	0.387				
b15	1.24 (0.89, 1.72)	0.197				
**b16**	**1.38 (0.94, 2.02)**	**0.100**	**1.52 (1.06, 2.16)**	**0.022**	**1.49 (1.04, 2.13)**	**0.029**
**b17**	**1.84 (1.23, 2.76)**	**0.003**	**1.96 (1.32, 2.91)**	**0.001**	**1.94 (1.30, 2.88)**	**0.001**
**b18**	**1.93 (1.16, 3.20)**	**0.011**	**1.96 (1.23, 3.12)**	**0.005**	**1.93 (1.21, 3.08)**	**0.006**
b19	0.91 (0.56, 1.47)	0.688				
b20	1.03 (0.69, 1.55)	0.881				

Model 1: 20 items enter the model using entrance method;

Model 2: 20 items enter the model using stepwise method;

Model 3: 8 significant items in model 2 added b6 and b7 using entrance method;

CFSS: Chinese frailty screening scale; OR: odds ratio; CI: confidence interval.

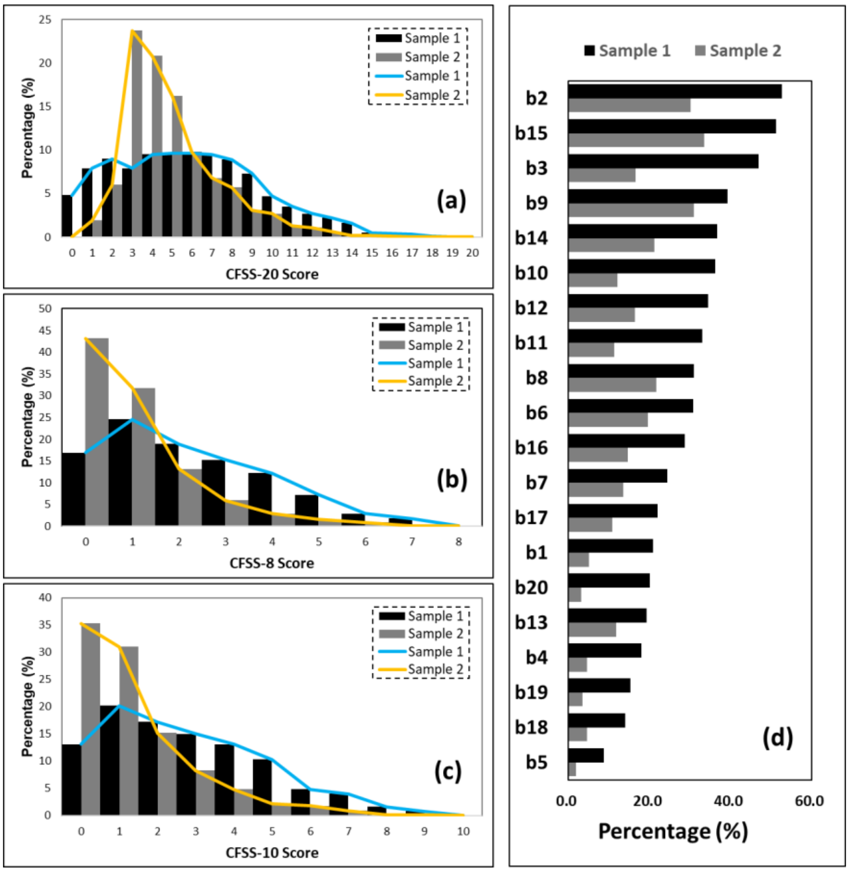

Figure S1. The distributions of the CFSS-20 (a), CFSS-8 (b), and CFSS-10 (c) score and prevalence of each item (d). CFSS: Chinese frailty screening scale.

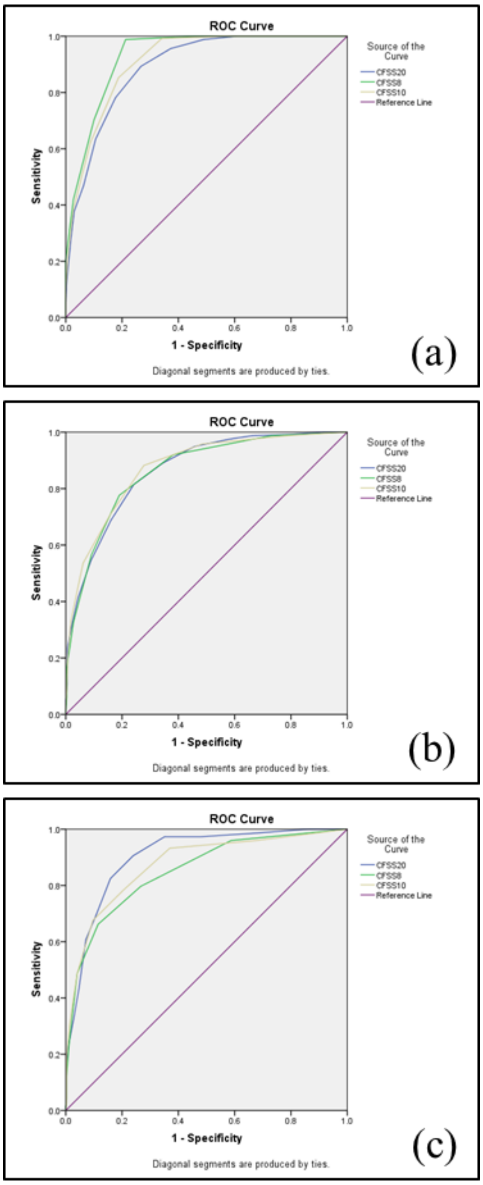

Figure S2. The comparison of AUCs of the CFSS-20, CFSS-8 and CFSS-10 take the FRAIL, TFI and FI as criteria (***sample 1***).

(a). Taking the FRAIL as criteria, the AUCs of the CFSS-20, CFSS-8, and CFSS-10 are 0.892 (95%CI: 0.872~0.911), 0.930 (95%CI: 0.916~0.945), and 0.911 (95%CI: 0.894~0.928), respectively; (b). Taking the TFI as criteria, the AUCs of the CFSS-20, CFSS-8, and CFSS-10 are 0.866 (95%CI: 0.839~0.892), 0.864 (95%CI: 0.836~0.891), and 0.874 (95%CI: 0.847~0.900), respectively; (c). Taking the FI as criteria, the AUCs of the CFSS-20, CFSS-8, and CFSS-10 are 0.904 (95%CI: 0.869~0.939), 0.851 (95%CI: 0.801~0.902), and 0.874 (95%CI: 0.826~0.921), respectively. CFSS: Chinese frailty screening scale; TFI: Tilburg frailty indicator; FI: frailty index; AUC: area under the curve.

Table S4. The results of the CFSS-10 predicting one-year disability and all-cause mortality without adjusting for comorbidity (***sample 2***)

Number of positive items	Disability (*n* = 1549)					All-cause mortality (n=2008)				
	n (%)	RR (95%CI)	*p* value	Adjusted RR (95%CI) [a, b]	*p* value	*n* (%)	RR (95%CI)	*p* value	Adjusted RR (95%CI) [a, c]	*p* value
0	119 (19.0)	reference		reference		3 (0.42)	reference		reference	
1	138 (26.2)	1.38 (1.11, 1.71)	0.004	1.18 (0.95, 1.46)	0.131	9 (1.45)	3.42	0.065	2.48 (0.65, 9.40)	0.181
2	63 (29.6)	1.55 (1.19, 2.02)	0.001	1.29 (1.00, 1.67)	0.054	12 (3.91)	9.24 (2.61, 32.74)	<0.001	5.48 (1.52, 19.81)	0.009
3	26 (25.5)	1.34 (0.93, 1.94)	0.121	1.24 (0.86, 1.78)	0.245	6 (3.59)	8.49 (2.12, 33.95)	0.002	4.33 (1.04, 17.94)	0.044
4	12 (26.7)	1.40 (0.84, 2.33)	0.196	1.10 (0.68, 1.78)	0.683	4 (4.17)	9.85 (2.20, 44.00)	0.003	3.69 (0.80, 17.04)	0.094
5+	16 (43.2)	2.27 (1.52, 3.40)	<0.001	1.55 (1.08, 2.23)	0.017	3 (2.78)	6.56 (1.32, 32.53)	0.021	2.10 (0.39, 11.21)	0.386
p for trend		< 0.001		0.032			< 0.001		0.251	

CFSS: Chinese frailty screening; RR: risk ratio, CI: confidence interval;
a. adjusted for age, sex, education and marital status;
b. using the Log-binomial model;
c. using the Poisson model.

Table S5. The results of the CFSS-8 predicting 1-year disability and all-cause mortality (***sample 2***)

Number of positive items	Disability (n=1549)					All-cause mortality (n=2008)				
	n (%)	RR	P value	Adjusted RR [a, b]	P value	n (%)	RR	P value	Adjusted RR [a, c]	P value
0	157 (20.7)	Reference		Reference		5 (0.58)	Reference		Reference	
1	141 (27.0)	1.31	0.008	1.15	0.124	10 (1.57)	2.72	0.068	2.06	0.198
2	42 (25.0)	1.21	0.211	1.02	0.884	14 (5.26)	9.13	<0.001	4.57	0.005
3	19 (31.2)	1.51	0.044	1.34	0.131	5 (4.17)	7.22	0.002	3.63	0.051
4	7 (30.4)	1.47	0.232	1.20	0.555	1 (1.69)	2.94	0.325	1.31	0.808
5+	8 (50.0)	2.42	<0.001	1.42	0.120	2 (3.39)	5.88	0.034	1.90	0.464
P for trend		<0.001		0.047			<0.001		0.234	

CFSS: Chinese frailty screening scale, RR: risk ratio;

a. adjusted for age, gender, education, marital status and comorbidity;

b. using the log-binomial model;

c. using the Poisson model.

Table S6. The results of the CFSS-8 predicting 1-year ADL and IADL disability (***sample 2***)

Number of positive items	ADL disability					IADL disability				
	n (%)	RR	P value	Adjusted RR [a, b]	P value	n (%)	RR	P value	Adjusted RR [a, c]	P value
0	28 (3.69)	Reference		Reference		157 (20.7)	Reference		Reference	
1	22 (4.21)	1.14	0.633	1.17	0.580	141 (27.0)	1.31	0.008	1.15	0.124
2	10 (5.95)	1.61	0.182	1.35	0.446	42 (25.0)	1.21	0.211	1.02	0.884
3	2 (3.28)	0.89	0.870	0.74	0.684	19 (31.2)	1.51	0.044	1.34	0.131
4	2 (8.70)	2.36	0.221	2.11	0.319	7 (30.4)	1.47	0.232	1.20	0.555
5+	2 (12.50)	3.39	0.076	2.50	0.220	8 (50.0)	2.42	<0.001	1.42	0.120
P for trend		0.080		0.258			<0.001		0.047	

CFSS: Chinese frailty screening scale, RR: risk ratio;

a. adjusted for age, gender, education, marital status and comorbidity;

b. using the Poisson model;

c. using the log-binomial model.

Table S7. The results of the CFSS-10 predicting 1-year ADL and IADL disability (***sample 2***)

Number of positive items	ADL disability					IADL disability				
	n (%)	RR	P value	Adjusted RR [a, b]	P value	n (%)	RR	P value	Adjusted RR [a, c]	P value
0	21 (3.36)	Reference		Reference		119 (19.0)	Reference		Reference	
1	24 (4.55)	1.36	0.309	1.39	0.282	138 (26.2)	1.38	0.004	1.18	0.136
2	12 (5.63)	1.68	0.153	1.51	0.268	63 (29.6)	1.55	0.001	1.28	0.062
3	4 (3.92)	1.17	0.777	0.74	0.626	26 (25.5)	1.34	0.121	1.19	0.357
4	2 (4.44)	1.32	0.705	1.13	0.866	12 (26.7)	1.40	0.196	1.13	0.628
5+	3 (8.11)	2.41	0.154	2.15	0.226	16 (43.2)	2.27	<0.001	1.57	0.017
P for trend		0.159		0.464			<0.001		0.035	

CFSS: Chinese frailty screening scale, RR: risk ratio;

a. adjusted for age, gender, education, marital status and comorbidity;

b. using the Poisson model;

c. using the log-binomial model.
